# Evaluation the stress distribution in root canals by 3D finite element analysis after the materials used in reattaching the vertically root fractured fragments

**DOI:** 10.1590/0103-6440202405833

**Published:** 2024-03-22

**Authors:** Senem Yiğit Özer, Salih Danişman, Törem Özer

**Affiliations:** 1 İzmir Demokrasi University, Faculty of Dentistry, Department of Endodontics, İzmir, Turkey; 2 Private Practice, Aydın, Turkey; 3 Turkish German Culture and Education Foundation School, ID 953, İzmir, Turkey

**Keywords:** Ceramic, acid etching, silane, electrical current, adhesive bond

## Abstract

The aim was to evaluate the effect of stress distribution on vertical, horizontal, and oblique forces on the tooth model after reattaching the fragments of the maxillary incisor with vertical root fracture (VRF) using different materials, by 3D finite element analysis (FEA). Tooth with a root canal, spongious, and cortical bone models were designed. VRF was modeled on a tooth with 4 different re-attachment models: Group 1: dual-cure cement (DC)+fiber reinforced composite (FRC), Group 2: DC+polyethylene fiber, Group 3: DC+glass fiber, and Group 4:DC. 100 N force was applied in 3 different directions. Maximum principal stresses (σmax) of dentin, and re-attachment materials were evaluated on colored images. The highest σmax values ​​were on the repair materials under vertical forces for Groups 1 and 4, respectively; Groups 2 and 3 showed similarity. The highest σmax values in repair materials under horizontal and oblique forces were observed in Group 3 however the lowest σmax values in repair materials under oblique and horizontal forces were observed in Group 1. The stress values ​​on repair materials gradually increased respectively starting from horizontal to vertical. As the elasticity modulus of the repair materials increased, the stress values ​​on root dentin increased. Through all force directions, except vertical forces, lower stress values were observed with FRC. The fracture resistance was bigger when using solely FRC or dual-cure resin cement in comparison to fiber-supported designs. Adding polyethylene fiber to re-restorations decreased stress values ​​compared to glass fiber addition. Therefore, when adding fibers, polyethylene fiber will be advantageous.

## Resumo

O objetivo foi avaliar o efeito da distribuição de estresse nas forças verticais, horizontais e oblíquas no modelo de dente após a recolocação dos fragmentos do incisivo superior com fratura radicular vertical (FRV) usando diferentes materiais, por meio da análise de elementos finitos (FEA) em 3D. Métodos: Foram projetados modelos de dentes com canal radicular, osso esponjoso e cortical. A FRV foi modelada em um dente com 4 modelos diferentes de reataque, como Grupo 1: cimento de cura dupla (DC) + compósito reforçado com fibra (FRC), Grupo 2: DC + fibra de polietileno, Grupo 3: DC + fibra de vidro e Grupo 4: DC. Foi aplicada uma força de 100 N em 3 direções diferentes. As tensões principais máximas (σmax) da dentina e os materiais de recolocação foram avaliados em imagens coloridas. Resultados: Os valores mais altos de σmax foram registrados nos materiais de reparo sob forças verticais para os Grupos 1 e 4, respectivamente; os Grupos 2 e 3 apresentaram semelhança. Os valores mais altos de σmax nos materiais de reparo sob forças horizontais e oblíquas foram observados no Grupo 3; no entanto, os valores mais baixos de σmax nos materiais de reparo sob forças oblíquas e horizontais foram observados no Grupo 1. Os valores de tensão nos materiais de reparo aumentaram gradualmente, respectivamente, começando da holizontal para a vertical. À medida que o módulo de elasticidade dos materiais de reparo aumentava, os valores de tensão na dentina da raiz aumentavam. Em todas as direções de força, exceto nas forças verticais, foram observados valores de tensão mais baixos com o FRC. Conclusões: a resistência à fratura foi maior quando se utilizou apenas FRC ou cimento resinoso de cura dupla em comparação com os designs com suporte de fibra. A adição de fibra de polietileno às restaurações diminuiu os valores de tensão em comparação com a adição de fibra de vidro. Portanto, ao adicionar fibras, a fibra de polietileno será vantajosa.

## Introduction

Vertical root fracture (VRF) is defined as a fracture originating from the inner side of the root surface and progressing to the outer surface along the long axis of the tooth ^1^. VRF is the third most common factor requiring tooth extraction after dental caries and periodontal diseases ^2^. It is known that the prognosis of teeth with VRF is hopeless, and therefore tooth extraction followed by implant placement has been the most recommended treatment option ^3^ ^,^ ^4^. However, it is difficult to create appropriate aesthetic conditions around dental implants, especially in the anterior region, and the need for soft tissue augmentation procedures is still debated ^5^ ^,^ ^6^. Atraumatic tooth extraction, re-attaching the fractured fragments extra orally using varied materials, and intentional replantation of the re-restored tooth an alternative treatment options recommended for VRF treatment ^7^. In addition, it is observed that implants may be associated with more complications and may require more postoperative care as compared to the natural tooth; hence, the argument may swing in favor of endodontics and tooth preservation ^8^.

There are in-vitro and in-vivo studies investigating the treatment of vertically fractured teeth ^9^ ^,^ ^10^ ^,^ ^11^ ^,^ ^12^. There are ethical problems in in-vivo research, as well as the difficulty of determining the behavior of tissues against applied forces ^13^. In addition, it is difficult to imitate the soft tissue that supports the tooth and standardization problems may be experienced in extracted tooth samples due to physical and anatomical differences ^13^. Variables such as the age of the teeth and the storage conditions after extraction may cause high standard deviations in evaluating the results ^14^. For these reasons, using virtual models, simulation modeling and finite element analysis (FEA) has become increasingly popular to limit the risks and costs of experiments to provide standardization ^15^.

Therefore, this study aims to evaluate the stress distribution in the tooth structure under loading forces, using FEA, according to the scenario of re-attaching the tooth fragments with VRF, using varied materials extra orally, and then intentionally replantation to the socket. The null hypothesis is that there will be no difference in terms of stress distribution under loading forces on reattached teeth using different repair materials.

## Material and methods

For editing and homogenizing the 3D network structure, an Intel Xeon ® R Central Processing Unit (CPU) with a 3.30 Gigahertz (GHz) processor, five hundred gigabytes (GB) Hard disk, and 14 GB Random access memory were used. Creating the 3D solid model and FEM operation, Windows 7 Ultimate Version Service Pack 1 operating system, Rhinoceros 4.0 (Seattle, WA, USA) 3D modeling software, VRMesh Studio (Virtual Grid Inc, WA, USA), and the analysis program Algor Fempro (ALGOR, Inc., PA, USA) was also used. After geometrically creating models using the VRMesh software, they were transferred to Algor Fempro software in Standard Tessellation Language (.stl) format to be ready for analysis.

**Figure 1:**
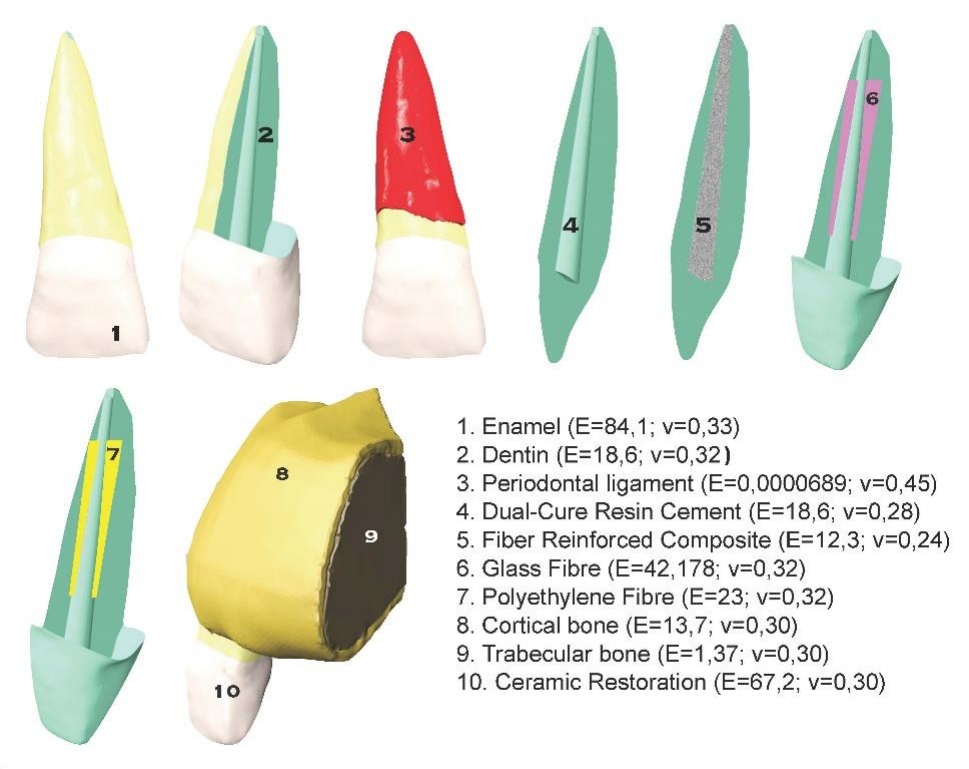
A solid virtual model of maxillary central incisor with vertical root fracture, elasticity, and Poisson ratio of each modeled structure 1. Enamel 2.Dentin 3. Periodontal ligament 4. VRF re-restored using dual-cure resin cement 5.VRF re-restored using fiber-reinforced composite 6. VRF re-restored using glass-fiber 7. VRF re-restored using polyethylene fiber 8. Cortical bone 9. Trabecular bone 10. Ceramic restoration

**Figure 2:**
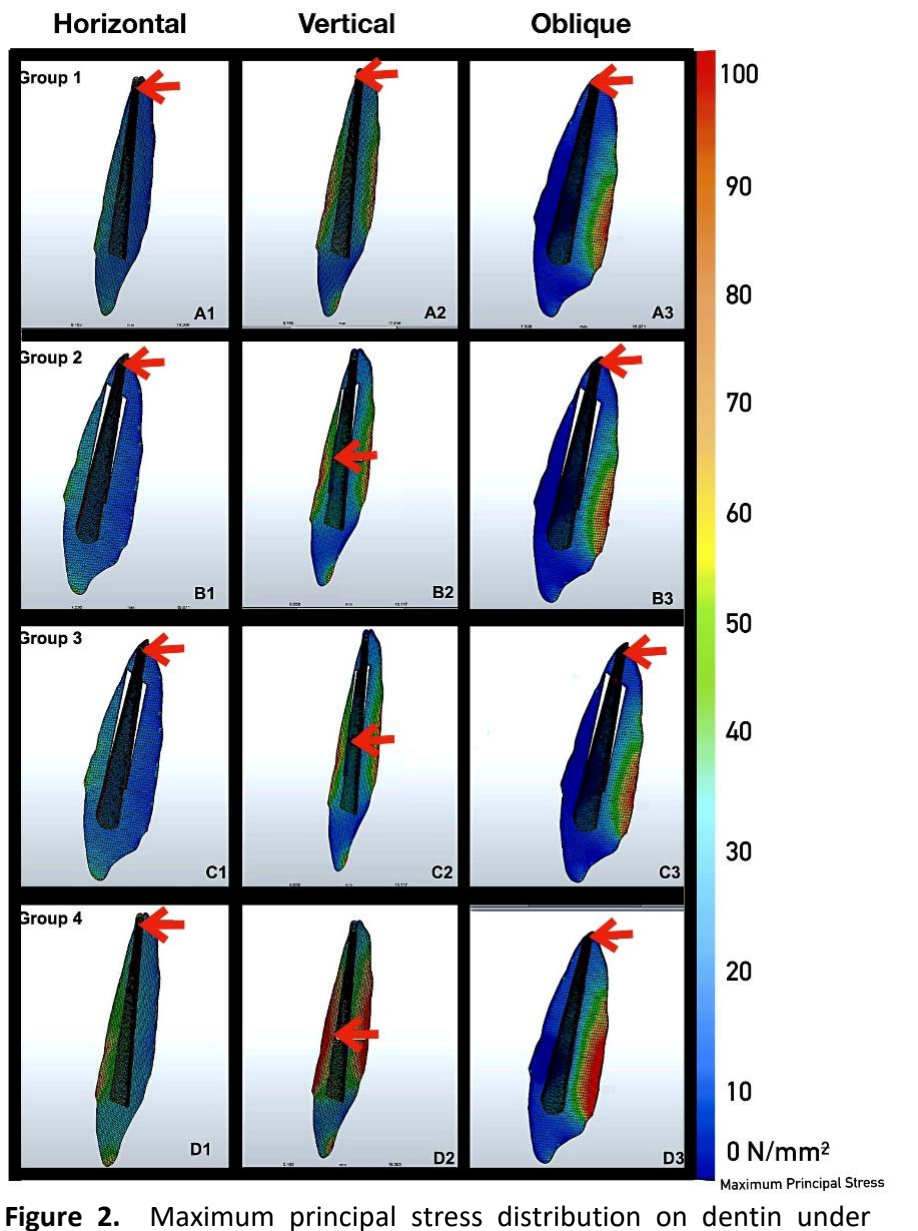
Maximum principal stress distribution on dentin under vertical, horizontal, and oblique forces. Colored areas indicate tensile stress. Red arrows show the areas where stress accumulates most intensely.

**Figure 3:**
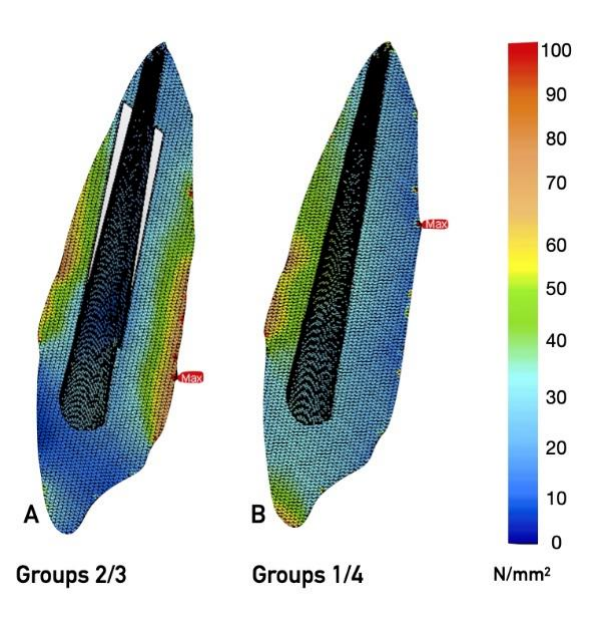
Maximum principal stress distributions calculated in corresponding finite element models. Blue to red colors represent stress values from low to high, respectively. Red arrows show the increased stresses and green areas show the increased stress within the root structure in the model simulating a re-restored vertical root fracture line. Both models are representative samples for A. Group 2, 3 B. Group 1, 4, respectively.

Maxillary anterior incisor tooth's enamel, dentin, periodontal ligament, bone tissue (cortical and cancellous bone tissue), dual-cured resin cement, fiber strips (polyethylene and glass), fiber-reinforced composite resin and porcelain restoration were modeled (Figure 1, 1 -10). A 3D finite element model was created in the Rhinoceros 4.0 program using the images of the incisor at different angles in the Wheeler dental atlas (Figure 1) ^16^. With the same software, a 0.25 mm thick periodontal ligament was modeled around the root (Figure 1,3). For cortical bone modeling (Figure 1,8), a 20x20x2 mm box was first modeled in Rhinoceros 4.0 (McNeill North America, Seattle, WA, USA) software. To mimic VRF, the crown was split into two halves, parallel to the long axis of the tooth, along the root in the frontal plane (Figure 1, 2).

The created VRF was modeled as reattached. Four separate groups were formed regarding re-restorations using different repair materials (Figure 1,4-7):

Group 1: Re-attached using dual-cured resin cement and fiber-reinforced composite (FRC).

Group 2: Re-attached using dual-cured resin cement and polyethylene fiber strip.

Group 3: Re-attached using dual-cured resin cement and glass fiber strip.

Group 4: Re-attached using dual-cured resin cement.

Group 1: The root canal cavity, which was split into two halves due to VRF, was modeled as re-attached by using both dual-cured resin cement and FRC (Figure 1,5). Ever X Posterior (GC, Tokyo, Japan) was used for FRC.

Group 2: The root canal cavity, which was split into two halves due to VRF, was modeled as re-attached by using both dual-cured resin cement and polyethylene fiber. The fiber was modeled as a rectangular prism located inside the root canal. The edge lengths of the prism were prepared in 0.4 mm thickness and 2 mm dimensions extending along 10 mm teeth. It was positioned at 3.5 mm from the end point of the root (Figure 1,7). Ribbond (Ribbond Incorporated, Seattle, WA, USA) was used as the polyethylene fiber. The cement thickness between the fractured roots was created to measure twenty-five microns (μ).

Group 3:The root canal cavity, which was split into two halves due to VRF, was modeled as re-attached by using both dual-cured resin cement and glass fiber. The fiber was modeled as a rectangular prism located inside the root canal. The edge lengths of the prism were prepared in 0.4 mm thickness and 2 mm dimensions extending along 10 mm teeth. It was positioned at 3.5 mm from the end point of the root (Figure 1,6). Stick-Net (StickTech Limited, Turku, Finland) was used as the glass fiber.

**Figure 4:**
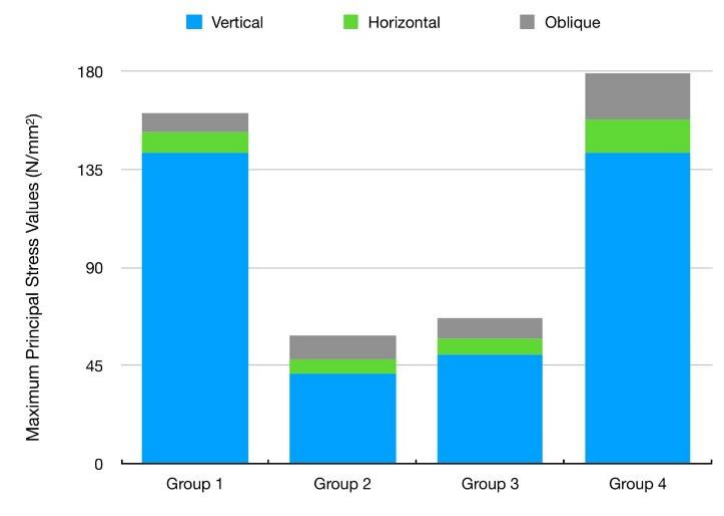
Maximum principal stress values on tooth dentine under horizontal, vertical, and oblique forces. There is a similarity between Groups 1 and 4 as in Groups 2 and 3; where fibers are used.

 Group 4: The root canal cavity, which was split into two halves due to VRF, was modeled as re-attached by using solely dual-cured resin cement (Figure 1,4). The cement thickness between the fractured roots was created to measure twenty-five microns (μ). Panavia F 2.0, a dual-cure resin cement; (Kuraray, Osaka, Japan) was used.

For all the tested groups, the root canal treatment model was not applied (which means endodontic treatment was not performed) since In this alternative treatment, root canals should be cleaned beforehand for the tight adaptation of the adhesive material into to root canal space ^12^

All groups were modeled as restored with full porcelain crown restoration. The boundary conditions of the crown restoration were prepared according to the crown prepared according to the Wheeler dental atlas ^16^. A 1 mm thick 135-degree (º) chamfer-designed step was formed, ending at the gingival level. The occlusal reduction amount was 2 mm, the axial reduction amount was 1 mm, and the axial wall angle was modeled as a prepared tooth of 6-8º. The thickness of the crown was determined as 2 mm on the cutting edges and 1 mm on the other regions. IPS Empress II (Ivoclar Vivadent, Schaan, Liechtenstein) was used for full porcelain crown restoration. A twenty-five μ-thick cavity was prepared for the cementation of tooth and crown restoration. RelyX ARC (3M ESPE, St Paul, USA) was used for cementation. As a result, an incisor model adapted to the maxilla was obtained (Figure 1,8-10). In Mesh Modeling, after the models were created geometrically with VRMesh (Virtual Grid Incorporated, Bellevue City, WA, USA) software, they were transferred to Algor Fempro software in. stl format so that they are ready for analysis. Material values ​​(modulus of elasticity and Poisson's ratio) describing their physical properties are given to each of the structures that make up the models (Fig 1). All models are considered linear, homogeneous, and isotropic. At the same time, it was accepted that the contact of the surfaces between the restoration/resin cement and the resin cement/tooth was 100 percent (%).

### Determination of loaded force and Boundary Conditions

The created model was fixed to have zero motion at each DOF (Degree of freedom) from the lower and posterior part of the jawbone. Then, a force of one hundred Newton (N) was applied to four separate groups from three different points. A force of 100 N was applied to the model in the direction parallel to the long axis of the tooth (F1-0º), perpendicular to the long axis of the tooth (F2-90º), representing the mastication force, in the oblique direction (F3-45º) in the palatal region. For each re-restored model, the maximum and minimum stress values that occur against the applied forces and their distribution were evaluated. The number of elements is 522994 and the number of nodes is 102688 for each model evaluated in the present study.

## Results

Figure 2 shows the σmax distribution of maximum principal stress values on dentin and repair materials.

The highest maximum principal stress values were on dentine under vertical forces for Groups 1 and 4 (Figure 2 A3-D3, 4), respectively, however, Groups 2 and 3 (Figure 2 B2-C2, 4) showed similarity. The highest σmax values ​​in the repair materials under horizontal and oblique forces were observed in Group 3 (Figure 2 C1-D1, C3-D3, Figure 3A, 4) however the lowest maximum principal stress values in the repair materials under horizontal and oblique forces were observed in Group 1 (Figure 2 A1-A3, Figure 3B, 4). Tensions were concentrated at the crown margin and cervical third of root dentin in all groups and under all loads. The stress values on the repair materials gradually increased respectively starting from horizontal, oblique to vertical directions.

## Discussion

In the in vitro study of Sen et al. ^9^, teeth with VRF were re-restored using dual-cured resin cement, polyethylene fiber, and glass fiber supports, and the re-attached models were subjected to fracture testing under vertical forces. It has been reported that the highest resistance to deformation occurred under using polyethylene fiber. Similarly, in the present study, the maximum principal stress values were found to be significantly lower in the models re-restored using polyethylene-reinforced composite (Group 2) and resin + glass fiber (Group 3) under vertical loads, which correlated with Sen et al.'s in vitro research. We may assume that insignificant differences regarding the groups evaluated may be due to using different trade firms with the same content, as stated in the conclusion part of the related study ^9^. Values may also be affected by different components such as the physiology and geometry of real teeth used in in-vitro research. In addition, the application of the materials of the experimental groups is different, affecting the results.

In addition, a case report suggesting a planned replantation treatment for VRF, in which a polyethylene fiber strip ^17^ had been used as support, was reported to have a successful treatment outcome for 3 years ^18^. Ozcopur et al. ^19^, investigating the fracture resistance of different post systems after rebonding teeth with VRF, stated that samples re-restored with polyethylene fiber strips produced more repairable fractures compared to glass fiber and metal-supported systems.

When the fibers used in the present study are compared, the modulus of elasticity gains importance. In addition, the chemical and physical properties of the fibers, and their interactions with dual-cured cement gain importance according to their production properties ^20^ ^,^ ^21^. It has been stated that the application of glass fiber with resin cement can reduce the fracture resistance of the residual monomer remaining in the resin cement ^21^. It also consists of the basic compound SiO_2_-Al_2_O_3_-CaO-MgO in glass fiber systems. ^22^. Boric oxide (B_2_O_3_) has been added to the glass fiber composition to increase the fiber's resistance to acidic environments. B_2_O_3_, which is a hard and glassy material in its pure form, increases the durability of the fiber or composite materials to which they are reinforced ^23^. However, due to the increase in the hardness of the material, the amount of stress transmitted to the dentin increases, and the possibility of fracture in the dentin increases. In the scanning electronic microscope research conducted by Vallittu ^22^, it was reported that the amount of B_2_O_3_ is higher in the Stick Net, which has been used, in the present study when compared to other glass fiber systems. Evaluating the results of FEM stress values, we may consider that the probability of a new fracture in the dentin in teeth with VRF is lower in polyethylene fiber than in glass fiber-restored re-attachments. As the reason for the lower stress values in dentin in the FRC composite structure, we can suggest that the FRC has a modulus of elasticity closer to dentin (Figure 1) and creates a more homogeneous monobloc structure compared to other groups assuming that there was no bond line reference when forming the attachments during FEM analysis.

Data reveal that VRF restored model using solely FRC or dual-cure resin cement shows the lowest risk of re-occurrence of VRF in root dentin. Polyethylene fiber produced lower maximum principal stress values ​​than glass fiber, as did the vertical force in fiber strip supported groups. In another in vitro study, polyethylene and glass fiber ribbon supports were compared ^23^. It was also stated that polyethylene fibers, which were found to be more successful, were more isotropic, more tightly packed, and more tightly oriented than glass fibers. It can be mentioned that the fiber shape and placement are also important for the applied fiber strip, apart from the elasticity modulus ^24^. There are also studies stating that the architecture of the fibers is more important than the elasticity modulus and the fiber shape ^25^. Karbharia and Strassler compared fiber reinforcements to a staple that holds the repaired surfaces together and prevents further fractures ^20^. In the present study, we can attribute the lower stress values of the polyethylene fiber-reinforced group to these reasons.

The hypothesis that there would be no difference in terms of stress distribution under different loading forces was rejected in teeth with VRF which are reattached using different repair materials was rejected. Major concerns and conclusions regarding the data may be as:

a. In all reattached models, the maximum principal stress values for repair materials occurred under vertical forces.

b. As the elasticity modulus of the repair materials increased, the stress values on the root dentin increased. Through all force directions, except vertical forces, lower stress values were observed in the models re-restored with fiber-reinforced composite. The models bonded with resin cement alone followed these values most closely. Since statistical data could not be generated with the FEA, the main idea that emerged because of the interpretations is that the resistance to fracture is bigger when using solely FRC or dual-cure resin cement during the re-restoration of VRFed teeth in comparison to fiber-supported designs.

c. Regarding the fiber-reinforced models, adding polyethylene fiber to re-restorations decreases stress values compared to glass fiber addition.

## Conclusions

The preference for polyethylene fiber will be advantageous during the re-restoration of VRF if adding fibers is desired.

Since all data was created and interpreted within the computer environment, the results are only in the form of mathematical data, and it is not possible to imitate clinical conditions exactly. The findings are only intended to provide a basis for future in vivo and in vitro studies using similar materials.
